# Samuel George Morton, M. D.

**Published:** 1851-07

**Authors:** 


### ARTICLE III.

### SAMUEL GEORGE MORTON, M. D.

It is with the greatest regret that we record the death of Samuel G. Morton, M. D., which took place in this city, on the 15th of May last. Although in feeble health for many years past, and evidently holding life by a very slight tenure, Dr. Morton was in the discharge of his ordinary professional duties up to the period of his last illness, which was of only four days' duration. His final attack was of an apoplectic nature; but he had long labored under considerable disease of the lungs and heart, as will be seen from the autopsy, appended to our notice, for the details of which we are indebted to Dr. Neill.

The death of Dr. Morton deprives our profession of one of its most distinguished members, and abruptly terminates a scientific career, which had become one of the brightest illustrations of our country. His reputation as a naturalist was diffused throughout the world, and we believe that no cotemporaneous American name was better known abroad than his. His loss, in every respect to be lamented, will be particularly felt in the department of natural hisiory, in which he was prosecuting the most interesting researches, when thus prematurely cut off, at the age of 52, in the prime of mental vigor and usefulness. In the relations of private life, no one ever conciliated more universal esteem and affection than Dr. Morton. He passed through the world literally without an enemy, beloved and respected in all circles, every where deservedly regarded as one of the most amiable and irreproachable of men.

Dr. Morton was a native of Philadelphia, but, we learn, passed a portion of his youth in New York. He pursued his medical studies in the University of Pennsylvania, and in the office of the late Dr. Parrish. After graduating in the University of Pennsylvania, he spent several years in Europe, and received a second degree from the University of Edinburgh.

Returning to his native city, Dr. Morton rapidly passed into extensive practice, and up to the time of his death, enjoyed a popularity second to none among the practioners of Philadelphia. To the literature of his profession he made numerous valuable contributions. In 1835 he edited Macintosh's Practice of Physic, which went through three subsequent editions; in 1833 he published an excellent original work on Consumption; and in 1849 an elaborate work on Anatomy.

Dr. Morton, was for many years engaged as a teacher of medicine, and always ranked among our best lecturers. He gave several clinical courses in the Philadelphia Almshouse Hospital, and was Professor of Anatomy in the original organization of the Pennsylvania Medical College.

With this assiduous and unfaltering devotion to strictly professional avocations, Dr. Morton combined an enthusiastic and earnest pursuit of natural history, which earned for him an European reputation, and gave many splendid results to science. His first scientific publication was a work on the *Fossils of the Cretaceous Group*, in which, we believe, he described every example that has been found in North America.

As far back as twenty years ago, he commenced collecting the materials, which he eventually embodied in the *Crania Americana*, the first work of the kind ever produced. This great work, published in 1839, immediately placed the author in the foremost rank among the cultivators of natural science, and was received throughout the world as a most valuable original contribution to ethnology. Its full importance is yet to be appreciated. At piesent it stands almost isolated ; and its real value can be developed only when other races of men are studied and tabulated on a similar plan, and a comparison of the averages afforded, enables us to determine the characteristic differences between the various races.

Dr. Morton spent many years upon the Crania Americana, and so careful was he to produce it free from inaccuracy, that after the elaborate tables had been nearly completed—upon Mr. George Combe's pointing out a slight error in the starting point from which the measurements had been made—a new series was at once commenced, and carried through on the corrected method.

The *Crania Egyptica* followed the *Americana*. The presence of the integuments, however, and the bituminized condition of the crania, prevented any definite series of measurements.

The subject of hybridity occupied much of Dr. Morton's attention in the latter period of his life. At the time of his death he was pursuing his inquiries in several interesting and hitherto unexplored channels, connected with this important branch of natural history; and he had already collected a vast number of facts, and reached the solution of many obscure and previously unnoticed points. In this course of investigation he was led into a close examination of the specific characters of the wolves of North America, and the result of the crosses between the different species of wolves and the imported dogs—a thread of inquiry which we know was developing most valuable conclusions in the highest walks of natural science.

In the midst of these absorbing scientific and professional labors, Dr. Morton found time to indulge a taste for poetry; and his occasional effusions show that he uniied a fine imagination, and refined appreciation of the beautiful, with his more solid powers and attainments. And all these noble intellectual qualities were graced with the crowning atrractions of a most unaffected bearing, the gentlest manners, and a genuine cordiality and kindness of disposition!

At the time of his death, Dr. Morton was President of the Academy of Natural Sciences, of which he had been a leading member for thirty years. He was also a fellow of the College of Physicians, of the American Philosophical Society and of numerous other learned societies, at home and abroad. The various associations with which he was connected in Philadelphia, united in every possible tribute of respect to his memory.

*Post Mortem Examination.*—On the day previous to his death, Dr. Morton was considered convalescent from an indisposition which his physicians had not supposed to be dangerous or alarming.

About the middle of the same day, a tendency to stupor was noticed, which gradually increased, and terminated in paralysis and death.

Present—Drs. Rodman, Beesley, Wistar, Pepper, McClellan and Neill.

*Head*—The symptoms immediately preceding death, directed attention particularly to the condition of the brain, it being supposed that effusion had taken place in that organ. The archnoid had lost its transparency, and no fluid was found in its cavity, but there was considerable serous effusion in the sub-archnoid cellular tissue.

The pia mater was very much congested, particularly on the left side, and from its vessels blood had been extravasated in several places. The basilar and vertebral arteries contained venous blood. Two small clots were found very nearly in similar positions upon either side, and rested upon the upper surface of the anterior lobe. It consisted of about one drachm of blood, and had assumed the form of the sulci, into which it had insinuted itself. The brain itself was large and symmetrical. Its substance was firm and natural in every respect. The choroid plexus was equally congested with the pia mater. Very little was found in the ventricles.

*Thorax.*—The pericardium contained the usual amount of serum, and presented no appearance of disease.

The heart was large and flabby, and its tissue was pallid and somewhat softened. The cavities generally were dilated; the parietes of those of the right side were particularly thin, and contained some fibrinous clots. The ostium venosum of the right side was enlarged to such a degree that it could not have been closed by the tricuspid valve. The mitral valve was much thickened by soft fibrinous yellow deposit.

The arch of the aorta was dilated and had some small patches of atheromatus matter deposited upon its internal surface.

The lefi lung was entirely useless for the purposes of respiration. It wvs contracted to a very great degree, and occupid but a small space in the upper portion of the thorax, where it was firmly bound down by the adhesion of the costal and pulmonarp pleura, by which the cavity of the pleura was entirely destroyed. Tne congestion of the lung was so great that it appeared dark colored and solidified, although it contained sufficient air to make it float in water.

The right lung, which was unusually large, was congested and adherent to the walls of the thorax by adventitious bands of an old formation. The cavity of the pleura contained about a half pint of bloody fluid, probably the result of post-mortem changes.

These adhesions of the left pleura, and the contraction of the left lung, were produced by an inflammatory affection of these organs, which occurred a few years since.

*Abdomen.*—The spleen was enlarged and very much congested. The tissue was so large and pulpy that it readily yielded to pressure by the finger.

The liver was natural· The alimentary canal was not examined.

It can readily be understood from the above facts, that the immediate cause of Dr. Morton's death was *meningeal apoplexy*, resulting from that disturbance of the circulation which was manifested by the engorgement of the blood-vessels of the brain and lungs, but which was produced by dilation of the heart.—*Phil. Med. Examiner*.

